# Effects of workplace upper extremity resistance exercises on function and symptoms of workers at a tertiary hospital: a randomized controlled trial protocol

**DOI:** 10.1186/s12891-022-05059-5

**Published:** 2022-02-05

**Authors:** Natália Claro da Silva, Flávia Pessoni Faleiros Macedo Ricci, Vinícius Restani de Castro, Alessandra Cristina Ramos de Lima, Ester R. do Carmo Lopes, Leonardo Dutra de Salvo Mauad, Karen A. Kawano Suzuki, Maria Eloísa de Oliveira Medeiros, Joyce Silva de Santana, Fernanda Ludmilla Rossi Rocha, Marisa de Cássia Registro Fonseca

**Affiliations:** 1 Rehabilitation and Performance Program, University of São Paulo, Ribeirão Preto, Brazil; 2 Paulista University, Ribeirão Preto, Brazil; 3 Ribeirão Preto Medical School, University of São Paulo, Ribeirão Preto, SP Brazil; 4 Department of General and Specialized Nursing, Ribeirao Preto College of Nursing, University of São Paulo, Ribeirão Preto, SP Brazil; 5 Department of Health Sciences, Rehabilitation and Performance Program, Ribeirao Preto Medical School, University of São Paulo, Av 3900, Ribeirao Preto –, Bandeirantes, SP 14049-900 Brazil

**Keywords:** Clinical trial, Strengthening exercise, Upper extremity, Prevention, Musculoskeletal pain, Health promotion, Ergonomics

## Abstract

**Background:**

Work-related musculoskeletal disorders (WRMDs) are often caused by inadequate use of the musculoskeletal system during work. Evidence suggests that multimodal intervention through exercises, massage, education, and ergonomic guidelines reduces pain and symptoms in the neck and upper extremities and help to prevent musculoskeletal disorders. The purpose of this study will be to assess the additive effectiveness of a specific and individualized workplace strengthening exercise program to an ergonomic guidance in reducing fatigue, pain and discomfort in the upper extremities and neck perceived by workers.

**Methods:**

This trial was designed according to the Consolidated Standards of Reporting Trials - CONSORT guidelines. Participants will be employees of a tertiary hospital, with any complaints of pain or discomfort in the upper extremities during the past 12 months, without clinical musculoskeletal diagnosis. 166 participants will be randomized into parallels groups as control and workplace exercises. The primary outcomes will be Numerical Pain Scale, isokinetic muscle strength of abduction and isometric handgrip strength. Secondary outcomes on discomfort, fatigue, work capacity and dysfunction will be assessed by QuickDASH, Patient Specific Functional Scale, Neck Disability Index, Need for recovery, Work Ability Index self-report questionnaires and FIT-HANSA performance test. The Ergonomic Work Analysis will be done by Quick Expose Check, RULA, REBA, RARME, ROSA and HARM risk assessment ergonomic tools. We will analyze the difference between baseline and 12 weeks of intervention by T test of independent samples (95% confidence interval, *p* < 0.05). Clinical significance will be analyzed by the minimum clinically important difference and effect size by Cohen index. The association between the variables will be analyzed by construct validity with the hypothesis of correlations between pain and muscle strength, strength and functionality and strength and fatigue.

**Discussion:**

Although studies have shown promise outcomes for workplace exercises as an available therapeutic resource used to minimize complaints of pain and discomfort related to work, the results of this study aim to bring evidence about the benefit of a specific resistance exercise as an effective modality to facilitate mechanisms of neuromuscular adaptations, with gradual and posterior hypertrophy in the later phases.

**Trial registration:**

(NCT04047056, https://clinicaltrials.gov/ct2/show/NCT04047056?term=NCT04047056&draw=2&rank=1) on Dec 03, 2020.

**Supplementary Information:**

The online version contains supplementary material available at 10.1186/s12891-022-05059-5.

## Background

Work-related musculoskeletal disorders (WRMDs) are a group of functional and mechanical disorders, often caused by overuse or inadequate use of the musculoskeletal system during work [1–3]. Muscles, tendons, fascia, nerves, joint and bones can be affected, and the result is fatigue, decreased performance at the workplace, temporary disability and absenteeism with economic consequences [4]. WRMDs are the second major cause of sick pay benefits in social security [5] and its prevalence has been increasing [1].

The most affected areas by WRMDs are neck, upper extremity, and low back [6–8]. Neck and upper extremity disorders can be divided into specific conditions with clear pathology and diagnosis, such as carpal tunnel syndrome, or non-specific conditions, which are defined by the location of symptoms, with unknown or imprecise physiopathology, such as neck strain syndrome [9].

Risk factors at the workplace related to neck and upper extremity symptoms include physical factors (harmful postures, repetitive tasks and static contractions), organizational factors (excessive work and absence of breaks), and psychosocial factors [6, 9–12]. Many tools have been developed and validated for the work ergonomics assessment. They aim to facilitate the survey of data regarding the various demands and workplace setting, postures assumed during professional activities, the perception of the worker regarding symptoms and muscle fatigue, and also to identify and classify the risk factors for the development of WRMDs [13–15].

In order to reduce WRMDs many companies have been investing more in prevention programs with a multidisciplinary approach for the promotion and maintenance of health, providing safer working conditions [16–18], adequate ergonomics [19] and workplace exercises [1, 9, 20]. Workplace exercises consist of specific activities performed during the working time. They usually have a short duration, with both preventive and therapeutic approaches, avoiding overloading or leading the employee to fatigue. It is a physical activity based on exercises to compensate repetitive movements, the absence of movement, or uncomfortable postures assumed during the working period [21].

To achieve more effective results with the implementation of workplace exercises, specific programs must be developed based on the objectives to be achieved. Some factors that are determinants of muscle performance must be taken into consideration, such as strength, power and endurance, together with the physiological adaptations to the exercises, which are influenced by intensity, exercise loads, volume, frequency and duration [22]. The resistance training is an effective method in the prevention and management of various disorders without distinction of sex. It is grounded on short term (6–8 weeks) mechanisms of neuromuscular adaptations, followed by gradual and posterior hypertrophy in the later phases (12–26 weeks) [23], leading to a balance and meeting individual’s needs and goals.

Recent studies aiming to evaluate the effectiveness of a physical exercise program performed in the workplace in reducing symptoms in the upper extremity and neck have shown promising results [1, 2, 4, 24–29]. Some randomized clinical trials have observed a reduction in pain and symptoms in the neck and upper extremity after the application of a multimodal intervention protocol composed by specific resistance exercises, muscle stretching, massage, educational and ergonomic guidelines within the work environment [2, 4, 20, 27–29].

Considering the aforementioned about previous studies, the aim of this study is to assess the additive effectiveness of a specific and individualized workplace strengthening exercises program to a protocol of ergonomic guidance in reducing fatigue, pain and discomfort in the upper extremity and neck of workers in a university hospital. Our hypothesis is that the group submitted to resisted exercises will demonstrate a decrease in musculoskeletal complaints and a better functional performance, in comparison to the group receiving only ergonomic guidance.

## Methods

### Trial design

**Fig. 1:**
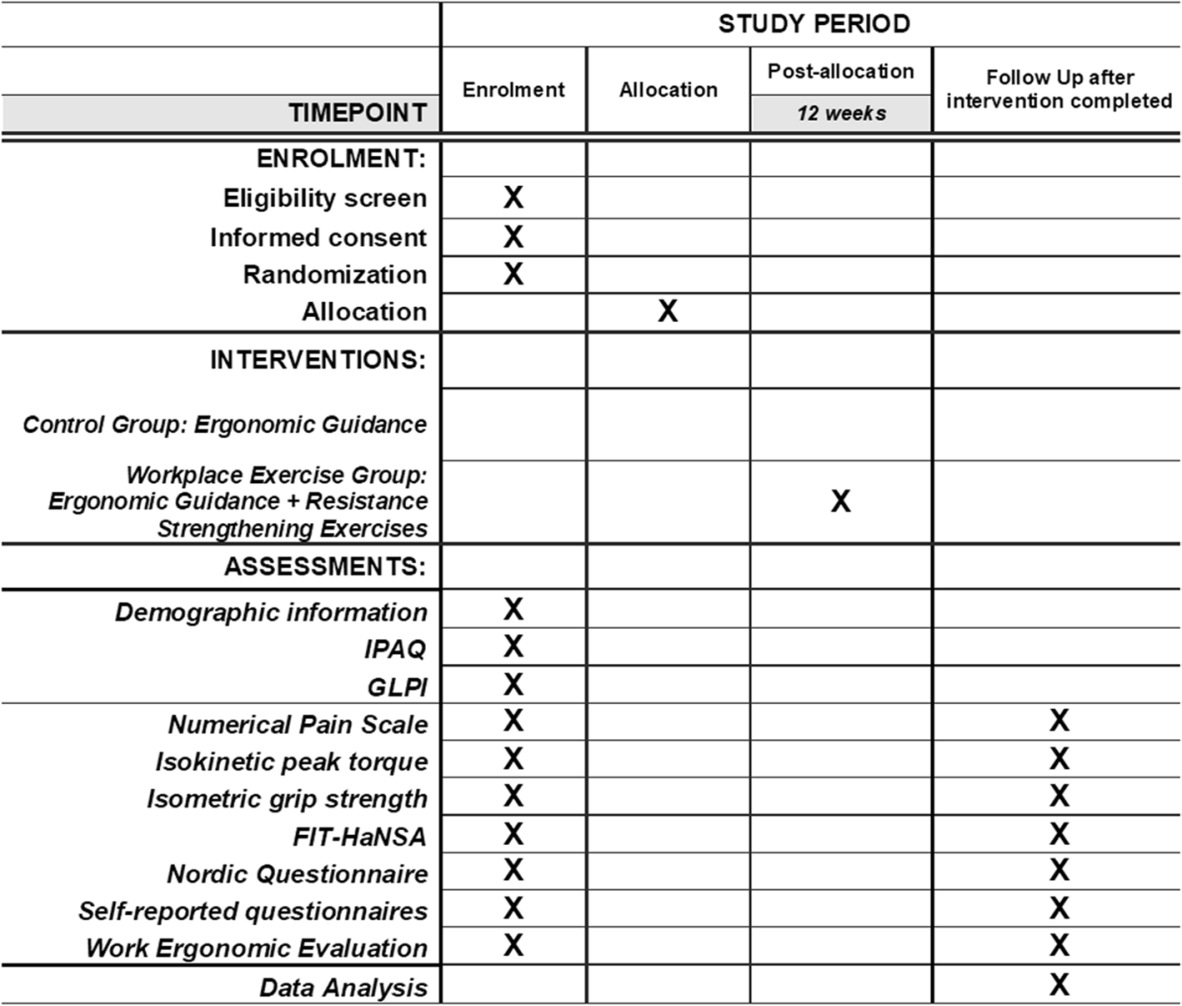
Study description

This will be a prospective randomized controlled trial with concealed allocation by using opaque, sealed envelopes consecutively numbered and included each group’s name and deviation from the intention-to-treat analysis [30]. The study was designed according to the Consolidated Standards of Reporting Trials - CONSORT guidelines [31] for randomized clinical trials and following the standard protocol items for randomized interventional trials (SPIRIT) [32]. Participants will be randomized to receive either ergonomic guidance alone or ergonomic guidance and workplace strengthening exercises (Fig. 1).

The study follows all ethical considerations set out in the Declaration of Helsinki and has received the approval from the Local Research Ethical Committee CAAE: 02658018.2.0000.5440. It was prospectively registered at www.ClinicalTrials.gov (NCT04047056).

### Participants

The participants of this study will be employees of a tertiary hospital, from any section, with any complaints of pain or discomfort in the upper extremity during the past 12 months. Potential subjects will be recruited by verbal invitation, posters and institutional cell phone messages. Inclusion and exclusion criteria are described in Table 1. All participants will sign a written informed consent form before participating.

**Table 1:** The Inclusion and Exclusion criteria description

Inclusion Criteria	Exclusion Criteria
• > 18 years old; • both sexes; • provide written consent; • workers who have musculoskeletal complaints in the past 12 months in the cervical region, shoulder, elbow, wrist or hand and/or fingers, without a clinical diagnosis; • workers who are not away from their professional activities.	• pregnancy; • congenital spinal abnormality and significant musculoskeletal deformities (such as amputation, dysmetria); • severe cervical spine disorders, postoperative conditions in the neck or upper limb region; • uncontrolled cardiovascular disease, cardiac arrhythmia, angina or related symptoms, and postural hypotension or other contraindication for physical exercise; • workers who engage in some form of regular exercise, which involves strengthening exercises and muscle resistance; • workers who have diagnosis of musculoskeletal dysfunction of the upper extremity and are undergoing physiotherapy treatment.

### Randomization

Employees who agree to participate will be randomized in a 1:1 ratio by a random allocation sequence by concealment envelopes in two parallel groups: control group (CG) and workplace exercise group (WEG).

### Blinding

Due to the nature of this study, it is not possible to fully blind the research participant or the clinician investigator providing the intervention. Randomization will be blinded for the outcome data collection and statistician assessors.

### Procedures for the experimental proposal of intervention

#### Control group

Participants in the CG will receive ergonomic guidance. The protocol will be composed by group lectures, digital handouts and specific ergonomic orientations and suggestions of low-cost changes in each participant’s workplace. The content of lectures and digital handouts will comprise information about what are WRMDs and how to prevent them and general posture and ergonomic guidelines, both for daily living and occupational activities.

#### Workplace exercise group

In addition to the ergonomic guidance, the WEG group will attend exercise sections 3 times/week, for 12 weeks. Exercise sections will take place inside the hospital, according to the workers’ availability during the breaks within working hours and will last 20 min. They will be divided as follows:

1. Five-minute warm-up movements (3 × 15 repetitions each);
2. Ten minutes of specific resistance strengthening exercises (3 × 10 repetitions each);
3. Five minutes of stretching and relaxing movements (3 × 30 seconds each).

Resistance strengthening exercises for cervical region and upper extremity will be performed with individually defined load and progression. The movements will be: flexion, abduction and shoulder elevation in the scapular plane; external shoulder rotation; “push up” on the wall; elbow flexion associated with pronation (concentric) and supination (eccentric) and manual grasping (Additional file 1: Appendix 1). Regarding the intervals between the movements, active recovery will be adopted to neutralize the effects of muscle fatigue. The criteria for interruption will be pain that incapacitates the execution of the movement, pain and /or discomfort or sensory alterations such as tingling and numbness.

**Fig. 2:**
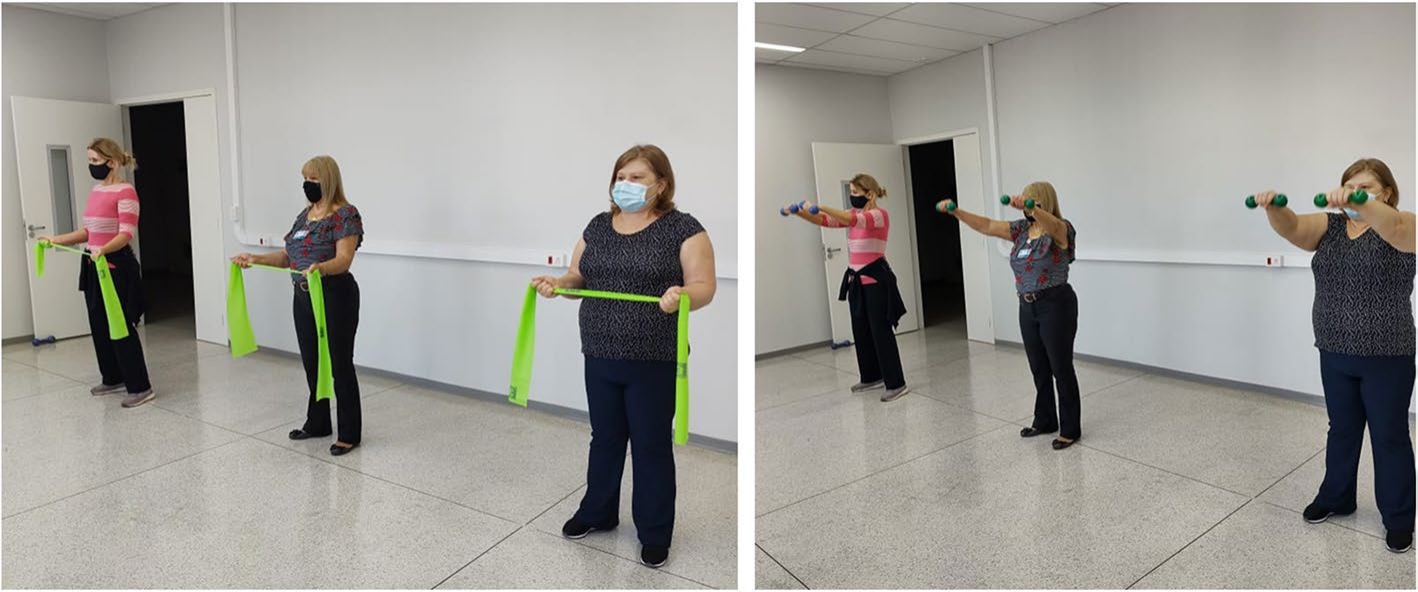
Small group exercises

Two physiotherapists with the collaboration of an undergraduate student will conduct the intervention. Sections will happen in small groups (Fig. 2), favoring social contact and integration among the employees, with the intention to improve the employee’s mood, as well as their adherence to the protocol.

#### Outcome measures

##### Demographic information

Participants will be interviewed and assessed for demographic and descriptive information including age, sex, weight, and height for the body mass index (BMI) calculation, history of smoking or alcoholism, dominance, occupation, work task (repetitive movements, and vibration), time of service, if any previous history of injury and symptoms perceived in the cervical region and upper limbs (intensity of pain, location and duration of the symptoms). Demographic information will be collected at the baseline examination only.

Additionally, the following self-reported questionnaires will be applied:

1. The International Physical Activity Questionnaire (IPAQ) short version, to characterize the sample as very active, active and irregularly active [33].
2. The Global Lateral Preference Inventory (GLPI) [34] to assess laterality and manual preference. The subject can fill out the activities according to his/her preference in the options: always left, majority left, indifferent, majority right, always right and I don’t know. Each of these answers has a different score. At the end, punctuation will be summed to generate the qualitative classification

##### Primary outcome measures

- Pain: assessed by the Numerical Pain Scale (NPS) [35].
- Mean isokinetic peak torque: workers will be submitted to the upper limb strength assessment in the isokinetic dynamometer Biodex System 4 Pro™, following all the calibration and use recommendations referred by the manufacturer in the manual “Biodex multi-joint system - pro setup/operation manual”. Based on Prentice and Voight (2003) [36], all movements will be performed at a speed of 60°/sec. The dynamometer positions will be based on the positioning guidance material, except for the scapular plane elevation that was adapted for this study, based on Kapandji (2000) [37]. The movement to be tested in isokinetic mode will be shoulder elevation in the plane of the scapula. The test will consist of 5 direct repetitions, with the volunteer being instructed to perform his maximum force during all repetitions, with an interval of 3 min between one segment and another [38]. Isokinetic tests will be done bilaterally to collect the parameters of the mean peak torque in Newton (N).
- Isometric palmar grip strength: will be measured by the JAMAR™ dynamometer, an instrument recommended by the American Society of Hand Therapists (ASHT) because of its reported reliability and validity [39].

##### Secondary outcome measures

- Fatigue: muscle performance will be assessed through the *The Functional Impairment Test-Hand and Neck/Shoulder/Arm (FIT-HaNSA)* [40]. This device assesses fatigue resistance during tasks that simulate gross motor functions of the upper limb, such as reaching and picking up objects at different heights and working sustained above the head [40]. The test consists of three tasks: 1) a shelf is placed at waist level and one 25 cm above it, 3 containers of 1 kg are placed on the lowest shelf; using the committed arm, the volunteer must place the 3 containers, from one shelf to the other, at a speed of 60 beats/minute controlled by a manometer; 2) a shelf is placed at eye level and one 25 cm below it. Volunteers are again instructed to use their affected arm to take the 3 containers from one shelf to another at a speed of 60 beats/minute. 3) A plate containing screws is fixed perpendicularly to the shelf. Volunteers are instructed to use both arms to tighten and loosen screws repeatedly. Each task will be performed only once, for a maximum of 300 s, or when the volunteer uses the criteria for stopping the test, which are: perform compensation with uncorrected trunk movements in up to 5 successive repetitions, interrupt the test due to extreme pain or fatigue or inability to complete 1 repetition of the movement within 2 beats of the metronome for 5 successive repetitions.
- Map of pain description: Nordic Questionnaire will be applied [41].
- Patient Specific Functional Scale (PSFS) [42], QuickDASH questionnaire [43], Work Ability Index (WAI) [44, 45], Need for recovery (NFR) [46] and Neck Disability Index [47] self-reported questionnaires will be applied.

Both primary and secondary outcome measures will be collected at baseline and after 12 weeks of intervention for all volunteers. Work ergonomic evaluation will be a complementary analysis, which integrates the scope of secondary outcome measures and also collected at baseline and after 12 weeks for both groups.

##### Work ergonomic evaluation

For the worker’s biomechanical overload and occupational exposure risk levels classification the following instruments will be used: the Quick Exposure Check (QEC) [48], Rapid Upper Limb Assessment (RULA) [49], Rapid Entire Body Assessment (REBA) [50], Musculoskeletal Risk Assessment Measurement (RARME) [51], the translated version of Hand Arm Risk Assessment Method (HARM) [52] and the Rapid office strain assessment ROSA [53]. They will be applied based on direct and indirect observation by photographic and videos records of the body and the upper limbs in the more critical situations reported by workers. Work ergonomic evaluation will be performed by 2 physiotherapists familiar with the instruments, blinded for the division of interventional groups.

## Data analysis

### Sample size calculation

The a priori sample size calculation was performed by GraphPad Statemate™, with power of 80%, effect size of 0.5 and alpha level of 0.05, based on the Numeric Pain Rating Scale and the isokinetic scores of the mean peak torque of the abduction movement in the scapular plane [54]. Seventh five participants will be needed per group. A total of 166 participants with pain or discomfort complaints of upper extremity who meet the inclusion / exclusion criteria and consent to participate will be included in the study, considering 10% sample size loss and analysis per intent to treat [30, 55].

### Statistical approach

An investigator will receive the encoded data and perform the statistical analysis entered into a database Excel spreadsheet. The Kolmogorov-Smirnov test will be used to verify the normality distribution of the data. Descriptive statistics (frequencies, means, standard deviation, confidence interval) will be used to analyze the sociodemographic characteristics of the participants. The significant statistical difference between the outcome groups will be analyzed by the T test of independent samples to compare the means at the baseline and after 12 weeks of intervention, using as outcome variables the numeric pain scale, the mean isokinetic scores of peak torque and work, the patient self-reported questionnaires, with 95% confidence interval, *p* < 0.05. Responsiveness values for interventions to changes in clinical significance will be defined to score the minimum clinically important difference [56]. The Cohen Index will be defined to calculate the effect size, considering 0.3 low, 0.5 moderate and 0.8 high effect [57]. The association between the variables will be analyzed through the construct validity by the hypothesis tests, which determine the convergent and discriminant validity [58, 59]. It is expected that there will be moderate inverse correlation between pain and muscle strength production, moderate correlation between strength production and functionality and strong to moderate correlation between strength production and fatigue endurance. The statistical program SPSS Statistics 20.0 will be used for all analyses [57, 60].

The confounding factors that could be accounted for in the statistical plan and potential to bias even with the randomization process are: occupations will be analyzed together for comparations between control and intervention groups, before and after the intervention but not in subgroups by level of activity load; and intention-to-treat analysis will be performed including data for the noncompliance volunteers, in exception for dropout cases due to loss of follow-up [61].

## Discussion

The WMSDs impair occupational activities, reducing productivity at work and increasing absenteeism. They also impact activities of daily living, reducing the quality of life in general. Besides, they generate economic consequences [4, 62]. Workplace exercise is an available therapeutic resource that has been widely used to minimize work-related complaints of pain and discomfort [1, 2, 9, 29]. Its benefits for workers’ health and for the company are already well known in some worker populations, but it is not yet well-established which exercise modalities would have the greatest benefit for workers.

Therefore, the purpose of this clinical trial is to evaluate the effects of a specific resistance exercise program associated with muscle stretching for the upper extremity and cervical region during the working day and compare them to just ergonomic guidance in reducing fatigue and complaints perceived by workers at a university hospital. We hypothesize that workers who receive the workplace exercise intervention will experience decreased pain, fatigue, dysfunction and better perceived long-term work capacity.

The results of this study will bring more evidence about the benefit of a specific resistance exercise program for workers with upper extremity complaints. Possible challenges for this study will include difficulties with participants recruitment and loss of participants for follow-up. To address these challenges, we will make the study widely known at the hospital in order to reach about all the professionals of the hospital in the different support areas such as maintenance, hygiene and cleaning and hospital nutrition, as well as employees in the administrative sector and health professionals.

The choice of the intention-to-treat analysis could reflect the reality of clinical practice in that workers often do not follow ergonomic or exercises instructions and sometimes take up a completely different approach from the one recommended. The limitation issue is that if the adherence is poor, then the results of the study may not be generalized well to workers who do comply with the intervention based on exercises in the intervention group and the ergonomic approach for both groups [63].

## Supplementary Information

**Additional file 1.**

## Data Availability

Not applicable.

## Abbreviations

WRMD: Work-related musculoskeletal disorders
CONSORT: Consolidated Standards of Reporting Trials
SPIRIT: Standard Protocol Items for Randomized Interventional Trials
NPS: Numerical Pain Scale
QuickDASH: Short version of Disability of Shoulder and hand questionnaire
PFSF: Patient Specific Functional Scale
NDI: Neck Disability Index questionnaire
NFR: Need for recovery
WAI: Work Ability Index
FIT-HANSA: The Functional Impairment Test-Hand and Neck/Shoulder/Arm
QEC: Quick Expose Check
RULA: Rapid Upper Limb Assessment
REBA: Rapid Entire Body Assessment
RARME: Musculoskeletal Risk Assessment Measurement
ROSA: Rapid Office Strain Assessment
HARM: Hand Arm Risk Assessment Method
IPAQ: International Physical Activity Questionnaire
GLPI: Global Lateral Preference Inventory
ASHT: American Society of Hand Therapists
CG: Control group
WEG: Workplace exercise group
