# Supplementary material for: Effects of workplace upper extremity resistance exercises on function and symptoms of workers at a tertiary hospital: a randomized controlled trial protocol
Source: BMC Musculoskelet Disord. 2022 Feb 5;23:119. doi: 10.1186/s12891-022-05059-5 (PMC8818236; doi:10.1186/s12891-022-05059-5)

Appendix 1. Resistance Strengthening Exercises Program

Shoulder Flexion

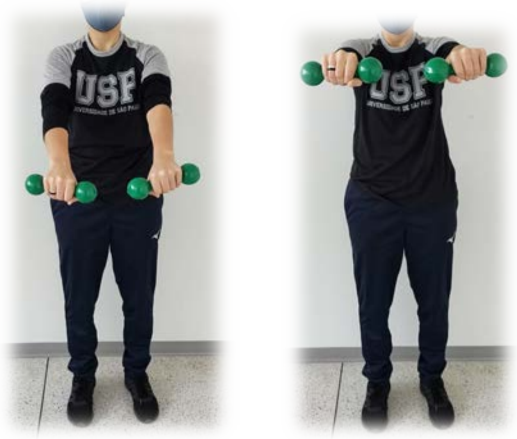

Shoulder Abduction

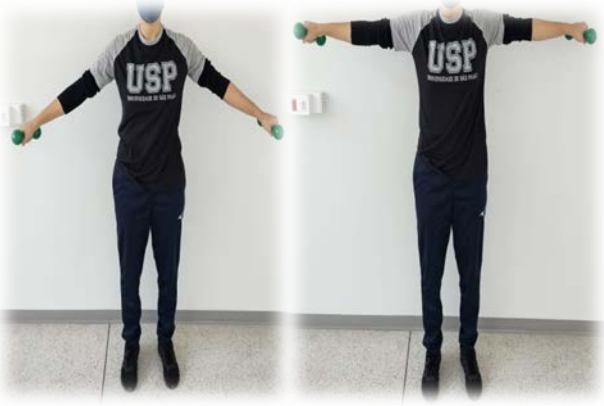

Shoulder Elevation – Scapular Plane

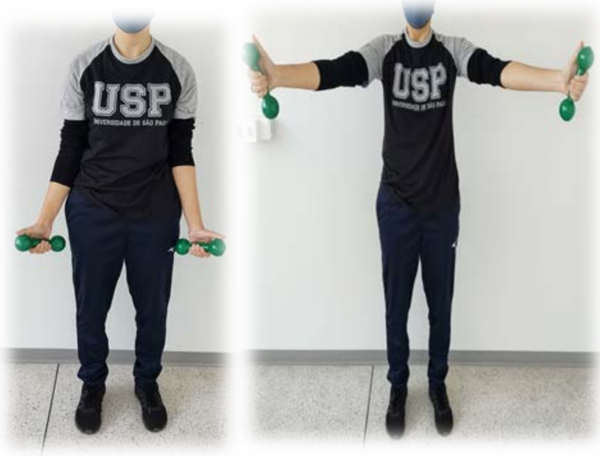

External Shoulder Rotation

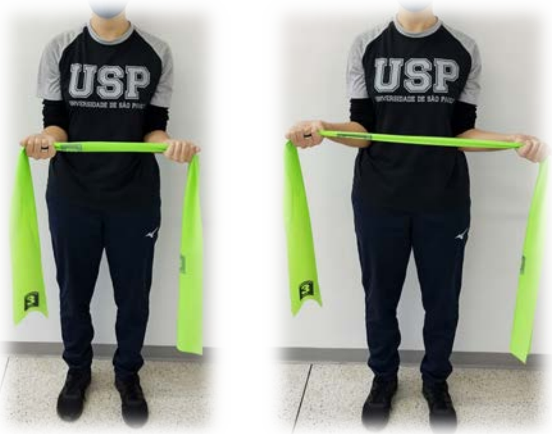

“Push up” on the Wall

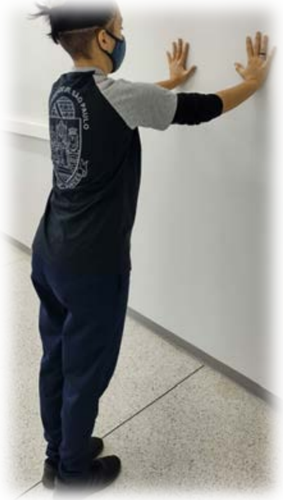

Elbow Flexion – Pronation/Supination

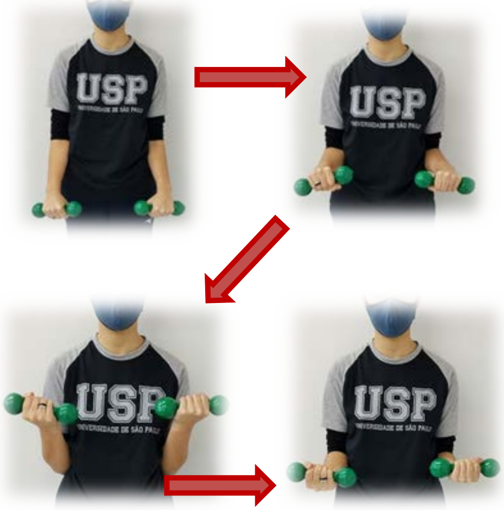

Supplement: Supplementary file 1 — Additional file 1. [file 12891_2022_5059_MOESM1_ESM.pdf]
